# Periosteal vascularization of the distal femur in relation to distal femoral osteotomies: a cadaveric study

**DOI:** 10.1186/s40634-016-0042-8

**Published:** 2016-02-01

**Authors:** J. A. D. van der Woude, R. J. van Heerwaarden, R. L. A. W. Bleys

**Affiliations:** Department of Orthopedic Surgery, Limb Reconstruction Center, Maartenskliniek Woerden, Polanerbaan 2, 3447 GN Woerden, The Netherlands; Department of Anatomy, University Medical Center, Utrecht, The Netherlands

**Keywords:** Distal femur, Distal femoral osteotomy, Valgus producing, Varus producing, Closed-wedge, Vascularization, Periosteal vessels, Cadaver, Surgical technique

## Abstract

**Background:**

The purpose of this study was to investigate periosteal vessels location as intra-operative landmarks in distal femoral osteotomies and focused on the branching pattern of the vascular supply of the medial and lateral femoral condyle, its constancy, and the relationship to the height of distal femoral osteotomies. Anastomoses of relevant vessels were studied to analyze the risk of vascular insufficiency after transection of landmark vessels.

**Methods:**

A human cadaver dissection study on the vascular supply of the medial and lateral side of the distal femur was conducted. Surgical dissection was performed in eight knees in total. Distances between the vascular supply and bony landmarks were calculated. Relation of the vascular structures to the transverse bone cuts of distal femoral osteotomies was described, as well as anastomoses of relevant vessels.

**Results:**

On the medial side of the distal femur the periosteum was primarily supplied by the descending genicular artery (DGA) in 87.5 % of the specimens. In the absence of the DGA, the superior medial genicular artery was the supplier. Vascularization took place through two constant branches, the upper transverse artery (UTA) and the central longitudinal artery. The UTA originated at a mean distance of 6.9 cm (range 5.9–7.9 cm) above the knee joint line. On the lateral side of the distal femur the superior lateral genicular artery was the main vessel. In all dissected knees it gave off the lateral transverse artery (LTA). The LTA originated at a mean distance of 6.9 cm (range 5.8–7.6 cm) above the knee joint line. Anastomoses between the UTA, LTA and the longitudinal arch of the femoral shaft were found that could prevent vascular insufficiencies after transection of the UTA and LTA.

**Conclusions:**

The vascular supply of the medial and lateral aspects of the femoral condyle is highly constant. Both the UTA, on the medial side, and the LTA, on the lateral side, can serve as a landmark for orthopedic surgeons in determining the height of the osteotomy cuts in distal femoral osteotomies. Transection of these landmark vessels during the osteotomy will not result in vascular insufficiency because of a collateral supply.

## Background

**Fig. 1:**
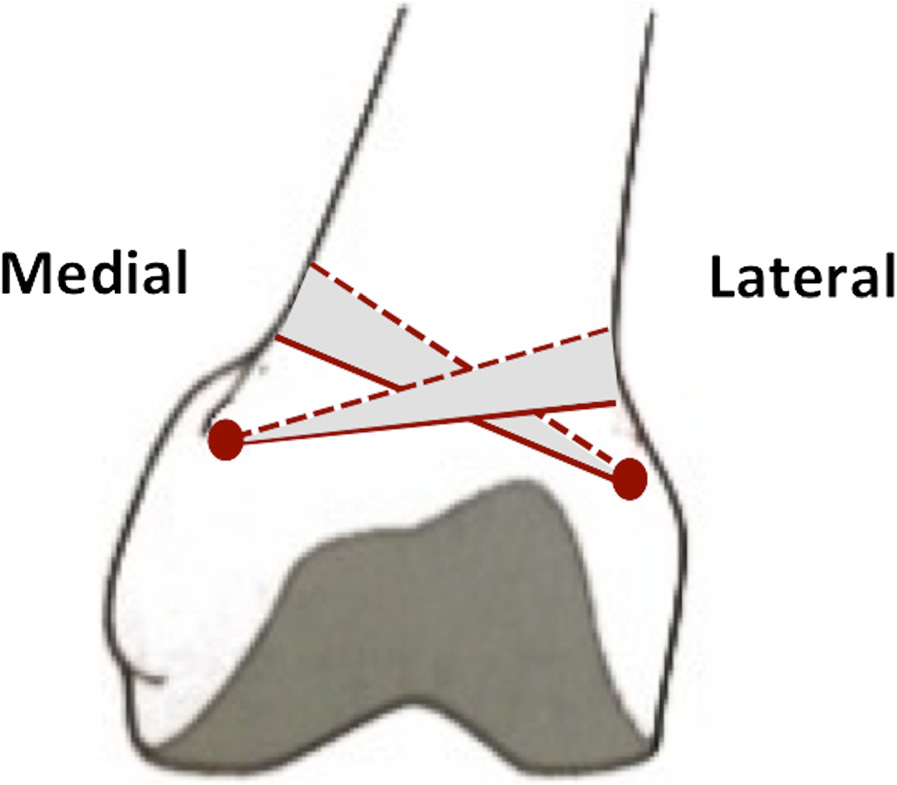
Schematic overview of the transverse osteotomy cuts in medial and lateral open- and closed-wedge osteotomies. The solid line represents the first transverse cut in open- and closed-wedge osteotomies. The dotted line represents the second transverse cut in closed-wedge osteotomies (height depending of pre-planned distance)

Osteotomies of the distal femur, for realigning a varus or valgus leg alignment in mono-compartment osteoarthritis and thereby unloading the degenerated part of the knee, are a well-established treatment (Hofmann et al. 2009). In new, improved osteotomy techniques bone cuts are made in the most distal metaphysical area of the femur, which is known for good bone healing capacity (Freiling et al. 2010; van Heerwaarden et al. 2013). In distal femoral open-wedge osteotomies the starting point of transverse osteotomy cuts lies approximately six and a halve cm above the knee joint line, medially as well as laterally, and approximately one cm proximal of the femoral condyles (Fig. 1) (Freiling et al. 2010; Visser et al. 2013; van Heerwaarden and Spruijt 2014). In distal femoral closed-wedge osteotomies the second transverse bone cuts are positioned in the area proximal to the open-wedge osteotomy (Fig. 1). In this area periosteal vessels have been observed medially as well as laterally that often need to be coagulated to prevent bleeding complications.

Osteotomies may cause bleeding complications not only as periosteal vessels are cut in the trajectory of the osteotomy bone cuts, but also when larger vessels near to the bone are not protected. Previous conducted cadaveric studies mainly have focused on high tibial osteotomy and the associated risk to the larger vessels (Darnis et al. 2014; Kim et al. 2010; Bisicchia et al. 2015; Tindall et al. 2006; Georgoulis et al. 1999; Ebraheim et al. 1998). With respect to the medial side of the distal femur, the vascular anatomy has been studied in the midvastus approach in total knee replacement surgery (Başarir et al. 2006; Scheibel et al. 2002). In contrast, discontinuing vascularity by cutting, suturing or coagulating vessels may cause vascular insufficiency. This has been studied with regard to the use of vascular bone grafts of the medial femoral condyle (Yamamoto et al. 2010; Iorio et al. 2011; Hugon et al. 2010; Huang et al. 2011). In addition, for both the medial and lateral side of the distal femur, the arterial supply was analyzed to find out if there are any differences in blood supply of the medial and lateral femoral condyles to explain the preponderance of osteonecrosis on the medial side (Reddy and Frederick 1998; Lankes et al. 2000). Furthermore, damage to small- and mediums-size vessels may be important to consider as a predisposing factor for delayed union and non-union of femoral osteotomies (Vena et al. 2013) and therefore it is important to know whether anastomoses are present preserving blood supply to the condylar area.

Specific literature on the vascular anatomy related to distal femoral osteotomies is scarce (Visser et al. 2013; Bisicchia et al. 2015). Visser et al. (2013) described a less invasive approach to the distal medial aspect of the femur in biplanar medial closed-wedge distal femoral osteotomy, which proved to be feasible and safe. Bisicchia et al. (2015) performed a cadaver study to assess the risk of vascular injury in realignment osteotomies, amongst them a medial closed-wedge osteotomy and a lateral open-wedge distal femoral osteotomy. However, the pattern of ramifications of the blood vessels which supply the femoral condyles, its variability and the topographical relation of these branches with the osteotomy height in distal femoral osteotomies have never been described. This study focused on the branching pattern of the vascular supply of the medial and lateral femoral condyle, its constancy, and the relationship to the height of the transverse osteotomy cuts in distal femoral osteotomies.

## Methods

Five left and three right fresh frozen lower limbs were obtained from eight human bodies. The specimens were derived from bodies who entered the Department of Anatomy of the University Center Utrecht through a donation program. From these persons written informed consent was obtained during life that allowed the use of their entire bodies for educational and research purposes. Each leg was amputated from the trunk about 10–15 cm below the hip joint, and the foot was amputated at the level of the conjoint fascia of the soleus and gastrocnemius muscle. The common femoral artery or superficial femoral artery was identified, cannulated, and flushed with normal saline until the venous outflow was clear.

### Dissection and sectioning

In all legs both the medial and the lateral structures covering the distal femur were dissected manually using regular sharp dissection techniques. The arteries could easily be recognized and dissected free from the surrounding structures. This resulted in an overview of the arterial branching pattern. All patterns and anatomic relationships to the surrounding soft tissues were photographed. Using ImageJ software (Image J 1.48, National Institutes of Health, USA) distances between the vascular supply of the medial and lateral femur condyle and bony landmarks were calculated. The chosen landmarks were: the knee joint line, the insertion of the adductor magnus tendon at the adductor tubercle (medial), the origin of the lateral collateral ligament (lateral), and the punctum maximum (most pronounced part) of both femoral condyles. Finally the relation of the vascular structures to the standardized heights of the medially and laterally started transverse bone cuts of distal femoral osteotomies was observed and described.

### Statistical methods

Descriptive statistics were used to report the distances. Measurements have been reported in centimeters (rounded to the first decimal); mean and ranges are provided. Statistical analysis was not performed due to the limited number of legs used.

## Results

### Dissection findings medial femoral condyle

**Fig. 2:**
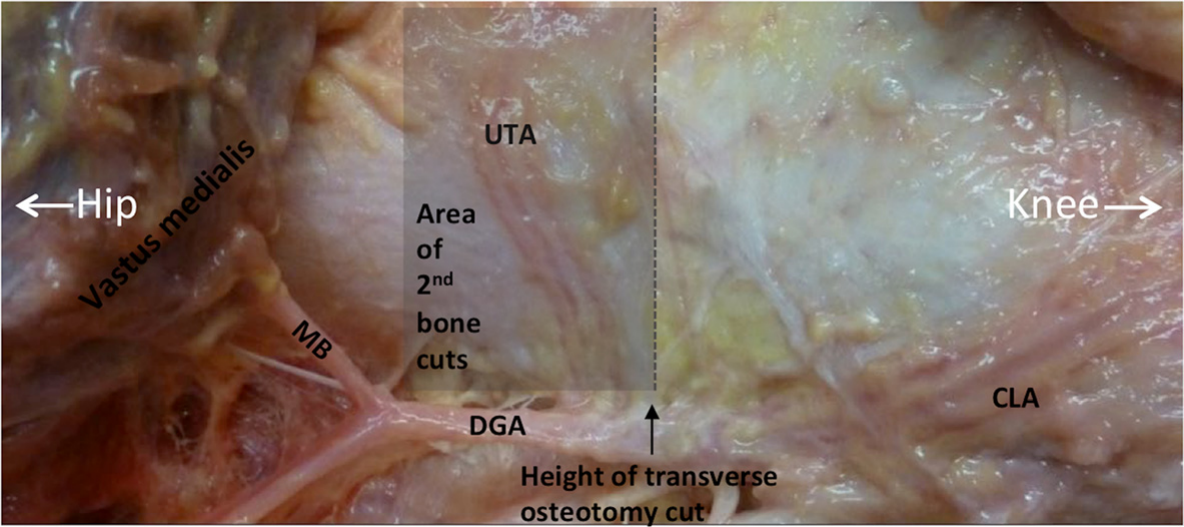
Medial side of a left distal femur with the typical branching of the descending genicular artery (DGA). Along its course the DGA gives off an anteriorly directed muscular branch (MB) to the vastus medialis, before the terminal branching in the upper transverse artery (UTA) and the central longitudinal artery (CLA). Each of the arteries is accompanied by two venae comitantes. The dotted line represents the height of the first transverse osteotomy cut (medial open- and closed-wedge distal femoral osteotomies) and the proximal grey zone is an example of the area were the second transverse bone cuts are positioned in medial closed-wedge distal femoral osteotomies (depending of pre-planned distance)

In seven of the eight dissected knees (87.5 %) the medial femoral condyle’s periosteum was primarily supplied by the descending genicular artery (DGA). The DGA originates from the superficial femoral artery, at a mean distance of 13.3 cm (range 10.8–15.1 cm) from the medial knee joint line. Hereafter it courses down to the adductor tubercle where it divides into two terminal branches: the upper transverse artery (UTA) and the central longitudinal artery (CLA) (Fig. 2). The UTA and the CLA were always present. The UTA originates at a mean distance of 6.9 cm (range 5.9–7.9 cm) above the medial knee joint line and descends anteriorly in an oblique manner. In Table 1 the distances between all landmarks and the UTA are given for each dissected knee. The CLA proceeds downwards in front of the adductor tubercle and of the medial collateral ligament. The superior medial genicular artery (SMGA) was present in all dissected knees and supplied the medial femoral condyle as the dominant vessel in one knee (12.5 %). The SMGA originated from the popliteal artery, at a mean distance of 6.0 cm (range 4.1–8.8 cm) above the knee joint line. After crossing from behind the adductor magnus tendon, it coursed anteriorly along the upper ridge of the medial femoral condyle.

**Table 1:** Distance between the UTA and LTA and the landmarks*

	UTA			LTA		
Knee number	Knee joint line	Insertion adductor Magnus tendon	Punctum maximum Medial condyle	Knee joint line	Origin lateral collateral ligament	Punctum maximum lateral condyle
1	7.5	1.3	4.7	7.6	2.0	4.5
2	5.9	1.0	3.8	6.2	1.7	3.7
3	7.0	1.6	3.5	5.8	1.0	4.3
4	7.6	1.0	4.6	6.8	1.8	4.6
5	6.0	1.1	4.9	7.4	1.8	5.0
6	7.9	1.8	4.5	7.3	1.8	4.7
7	6.8	1.6	3.6	6.9	1.8	4.7
8	6.7	1.2	3.9	7.2	1.8	4.0
Mean (range)	6.9 (5.9–7.9)	1.3 (1.0–1.8)	4.2 (3.5–4.9)	6.9 (5.8–7.6)	1.8 (1.0–2.0)	4.5 (3.7–5.0)

*In centimeters

**Fig. 3:**
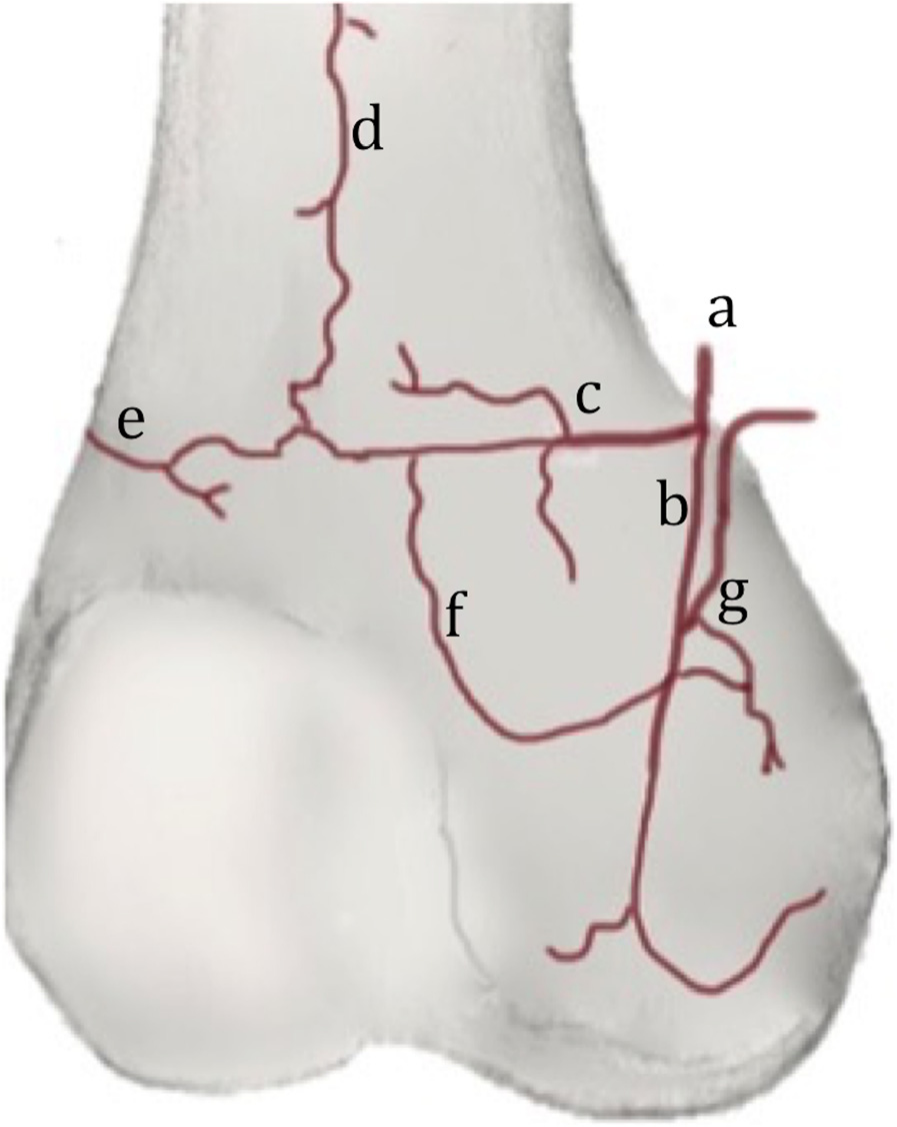
Anastomoses of the arterial vascularization of the femoral condyle (anteromedial view of a right knee). **a** Descending branch of the descending genicular artery (DGA). **b** Central longitudinal artery (CLA). **c** Upper transverse artery (UTA). **d** Longitudinal arch of the femoral shaft. **e** Lateral transverse artery (LTA). **f** Anastomotic arch of the medial condyle. **g** Branch of the superomedial genicular artery

The terminal ramifications of the UTA and CLA anastomose with the longitudinal arch of the femoral shaft (Fig. 3). The terminal branches of the SMGA anastomose with those of the DGA. In the case where the DGA was absent, the upper transverse and the central longitudinal artery originated from the SMGA. The longitudinal arch of the femoral shaft was identified in each dissected knee. This longitudinal arch originates from the superficial femoral artery, at a distance of 13.4 cm (range 7.7–16.9 cm) above the knee joint line, proximal to the DGA branch takeoff.

### Dissection findings lateral femoral condyle

**Fig. 4:**
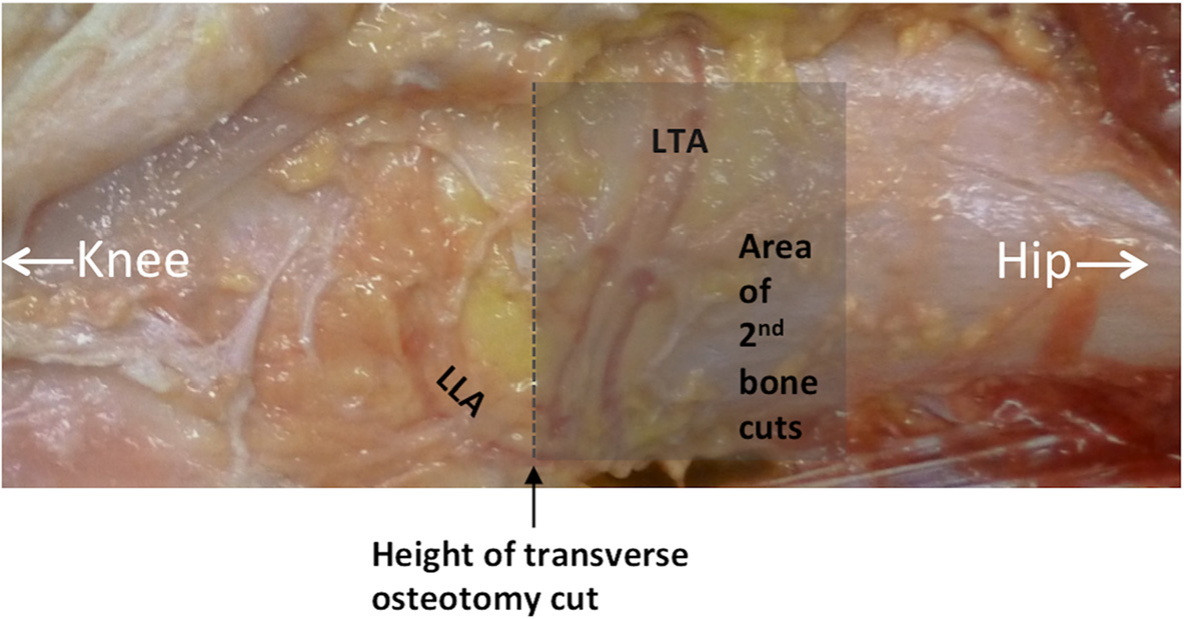
Lateral side of a left distal femur with the branching of the superior lateral genicular artery. The lateral transverse artery (LTA) is the transversely travelling artery and is accompanied by two venae comitantes. The LLA is the lateral longitudinal artery. The dotted line represents the height of the first transverse osteotomy cut (lateral open- and closed-wedge distal femoral osteotomies) and the proximal grey zone is an example of the area were the second transverse bone cuts are positioned in lateral closed-wedge distal femoral osteotomies (depending of pre-planned distance)

The periosteum of the lateral femoral condyle was supplied by the superior lateral genicular artery (SLGA) in all dissected knees. The SLGA originates from the popliteal artery, at a mean distance of 6.2 cm (range 3.9–8.7 cm) above the lateral knee joint line. Conversely with the SMGA, it courses along the upper ridge of the lateral femoral condyle, where it forms terminal branches. One of them travels transversely, being the lateral transverse artery (LTA) (see Fig. 4). The LTA was in all dissected knees present. The LTA originates at a mean distance of 6.9 cm (range 5.8–7.6 cm) above the lateral knee joint line and descends anteriorly in an oblique manner. In Table 1 the distances between all landmarks and the LTA are given for each dissected knee. The terminal ramifications of the LTA anastomose with the longitudinal arch of the femoral shaft and with the UTA (Fig. 3).

### Relation of the vascular structures to location of osteotomy cuts

A constant branch pattern of the vascular supply of the medial and lateral femoral condyle, related to the height of the transverse osteotomy cuts in distal femoral osteotomies was observed. Each of the arteries is accompanied by two venae comitantes, which make them easily recognizable (see also Figs. 2 and 4). The UTA and LTA are located in the area 6.5 cm proximal to the medial and lateral knee joint line respectively, where transverse cuts for medial and lateral open-wedge and closed-wedge osteotomies are positioned. In osteotomy surgery of the distal femur the UTA and LTA can serve as consistent landmarks for transverse osteotomy height position.

## Discussion

The most important finding in this study is that the vascularization of the medial and lateral aspect of the femoral condyle is highly consistent and characterized by anastomoses between the UTA, the LTA and the longitudinal arch of the femoral shaft. Moreover, the height of both the UTA (on the medial side) and the LTA (on the lateral side) are in line with the height of the osteotomy cuts in distal femoral osteotomies and thus can serve as an intra-operative landmark for orthopedic surgeons.

Intra-operative landmarks are an important help for the surgeon, not only in standard “open” approaches of the distal femur (Freiling et al. 2010; van Heerwaarden and Spruijt 2014) but even more in mini-invasive approaches (Visser et al. 2013; Farouk et al. 1998, 1999). Especially in the latter approach only a keyhole view is present of the bone area for positioning of the osteotomy cuts. Precise positioning of the transverse osteotomy cuts is crucial to the success of the surgical technique and a consistent landmark on the bone helps the surgeon in addition to fluoroscopic assistance. In this study it was found that if the guiding wires for the osteotomy cuts are positioned immediately distal to the UTA and LTA the optimal medial, respectively, lateral starting points for open-wedge osteotomies are used. The second transverse bone cut used in closing-wedge techniques will be started more proximal at a pre-planned distance from the first osteotomy cut. In both osteotomy techniques it is safe to coagulate the landmark-vessels to prevent bleeding as the anastomoses prevent vascular insufficiency of the medial and lateral femoral condyle.

To our knowledge, in the past only one study was conducted that described the presence and distances to the knee joint of the UTA. No earlier work describing the presence and distances to the knee joint of the LTA has been published yet. Hugon et al. (2010) described the UTA in 100 % of the knees (16) they dissected. In those 16 knees the mean distance between the origination of the UTA and the knee joint line averaged 7.2 cm, with a minimum distance of 5.4 cm and a maximum distance of 8.9 cm. This is in line with our average of 6.9 cm, minimum of 5.9 cm and maximum of 7.9 cm. In the current study, the DGA was present in 87.5 % of the dissected knees. This is in line with earlier reports, which state a presence of 85–100 % (Yamamoto et al. 2010; Hugon et al. 2010; Dubois et al. 2010).

In 1950, Rogers and Gladstone (1950) were the first to review the intra- and extra-osseous blood supply of the distal femur. They stated that the medial condyle arteries originate from the DGA and the SMGA and those were richly anastomosing and ultimately perforating the cortex to vascularize the bone. Later, Shim and Leung (1986) confirmed this with a microangiographic study. Hugon et al. (2010) also described many anastomoses between the branching of the periosteal vascularization of the medial femoral condyle with the SLGA, the muscular branches of the vastus intermedius and the longitudinal arch of the femoral shaft. In addition, they even noticed that they found numerous arteries entering the bone posteriorly and connecting with the periosteal arteries, without forming any form of watershed line. The findings of these studies are in line with the anastomoses we described between the UTA, LTA, and the longitudinal arch of the femoral shaft. Regarding risk areas for vascular insufficiency, our study did not focus on this topic. However, in literature this has been extensively described. Reddy and Frederick (1998) reported a relative watershed region in the anterior portion of the medial condyle. Furthermore, they stated that subchondral bone of the lateral femoral condyle is well supplied and has a richer circulation with more collateral supply than the medial side. In a similar study Lankes et al. (2000) found the region of the femoral insertion of the posterior cruciate ligament (anteriorly in the intercondylar fossa) to be avascular. They did not describe the relative watershed as mentioned by Reddy and Frederick. Vascular insufficiency manifested i.e. by osteonecrosis has not been cited in the literature as a postoperative occurrence after femoral osteotomies (Vena et al. 2013) nor as a complication after corticopertiosteal vascularized grafting from the medial femoral metaphysis (Doi et al. 2000; Del Pinãl et al. 2007; Sakai et al. 1991).

A limitation of this study is the limited amount of knees that were dissected and investigated. This may have led to a type II error. However, the relatively small population size of *n* = 8 is not unusual in labor-intensive anatomic research (Bleys et al. 1996; Toorop et al. 2013). The findings were consistent and therefore the sample size was sufficient to meet the purpose of this study. Furthermore, we found a high correlation with the results of previously reported studies on distal femur vascularization. Another limitation is the possible length variability of each cadaver. One of the possibilities to address this issue would be to use the relative distance of the lower limb or femur for interpretation. However, the legs were already amputated prior the dissection and sectioning, so this data was not available. We did not use any in- or exclusion criteria for the knees, and the dissected knees population in this study did not contain severely arthritic knees. Relationships between arteries and bony landmarks can change in osteoarthritis of the knee (Eriksson and Bartlett 2010). However, these differences are reported to be minimal in the sagittal and coronal plane (0.8–1.6 mm) and therefore its clinical implications are questionable (Lee et al. 2014). So, even if there had been knees included with a high-grade osteoarthritis, this would not have biased the results of our study.

## Conclusions

In conclusion, in this study the vascularization of the medial and lateral aspect of the femoral condyle was found to be highly consistent. Both the UTA, on the medial side, and the LTA, on the lateral side, can serve as a landmark for orthopedic surgeons in determining the height of the transverse cuts in open- and closed-wedge distal femoral osteotomies. The UTA and LTA can be cauterized in a safe way, and bone cuts can be made at the level of these vessels since there are many anastomoses in the periosteal vascularization of the medial and lateral femoral condyle.
